# Relationships between Motor Competence, Physical Activity, and Obesity in British Preschool Aged Children

**DOI:** 10.3390/jfmk3040057

**Published:** 2018-11-21

**Authors:** Charlotte J. S. Hall, Emma L. J. Eyre, Samuel W. Oxford, Michael J. Duncan

**Affiliations:** Centre for Sport, Exercise and Life Sciences, Coventry University, Coventry CV1 5FB, UK

**Keywords:** physical activity, motor competence, preschool, children, obesity, MVPA

## Abstract

Background: This cross-sectional study aimed to examine associations between motor competence, physical activity, and obesity in British children aged three to five years. Method: Motor competence (MC) was assessed using the Test of Gross Motor Development-2. Physical activity (PA) was assessed using triaxial wrist-worn accelerometers. Children were assessed on compliance to current PA recommendations of ≥180 min of total PA (TPA) and ≥60 min of moderate-to-vigorous PA (MVPA) for health benefits. Associations were explored with Pearson’s product moments and weight-status, and sex-differences were explored with independent *t*-tests and chi-squared analysis. Results: A total of 166 children (55% males; 4.28 ± 0.74 years) completed MC and PA assessments. Associations were found between PA and MC (TPA and overall MC, TPA and object-control MC (OC), MVPA and overall MC, and MVPA and OC). This study suggests that good motor competence is an important correlate of children meeting physical activity guidelines for health.

## 1. Introduction

Childhood obesity is a serious public health challenge of the 21st century, with enduring adverse consequences for health outcomes. Over 42 million under-fives are estimated to be overweight or obese [1,2], and current predictions are 70 million children will be overweight or obese by 2025 [1]. Elevated body mass index (BMI) is a major risk factor for non-communicable diseases such as cardiovascular disease, diabetes, and some cancers [2]. Overweight children have an increased risk of adulthood obesity, premature death, and disabilities in adulthood, resulting in prevention of childhood obesity being considered as a public health priority [2,3,4].

Overweight children are less physically active than their healthy weight peers [5], and therefore improving participation in physical activity (PA) is seen as a key preventative focus [6]. Current physical activity (PA) guidelines [2,7] recommend children under 4 years complete ≥180 min of total PA, including light and moderate-to-vigorous PA (MVPA) (total PA, TPA), and children aged 4–17 years complete ≥60 min MVPA per day. In children, MVPA has been shown to play a key role in health, particularly when related to weight-status [8,9]. However, it is widely reported that children under five years are not sufficiently active [8,10,11,12,13].

Studies in American and German children using uniaxial accelerometry have reported that children are not meeting either PA guidelines [10,11]. British preschoolers have similarly been reported to not meet current PA guidelines, although weight status is reported to not influence the amount of PA undertaken by preschoolers [8,12,13]. Prior work has reported that British preschoolers achieved on average 38.6 min of MVPA and 96.7 min of TPA per day [8]. In two related studies, examining preschoolers from the same geographical area as O’Dwyer et al. [8], similar magnitudes of PA have been reported [12,13]. No significant sex or weight-status differences between MVPA completion rates were reported in this prior work [8,12,13]. However, the PA cut-points used by O’Dwyer et al. [8,12,13] were validated in American preschool children [14]. Use of cut-points derived from American preschoolers to assess PA in a British population may not fully capture the PA behavior of children, due to the different social and economic environments in which PA takes place in the U.S. compared to the U.K. Conversely, other research has reported that 95.6% of British preschoolers completed TPA recommendations for health. Similar to the aforementioned studies by O’Dwyer et al. [8,12,13], Barber et al. [15] used cut points which were validated in American preschoolers [16] despite investigating children from Bradford [15]. Preschool-based approaches that decrease sedentary behavior and increase PA may aid in combating the epidemic of juvenile obesity. However, to avoid geographical and demographical bias on PA, using British validated PA cut-points designed for British children is crucial to better quantify PA, and would establish valid current PA patterns.

According to Stodden et al. [5], motor competence (MC) is a precursor to PA, and learning to move is necessary for participation in PA. Fundamental motor skills (FMS) develop during childhood, form the foundation for lifelong PA, and are conceptualized as comprising locomotor skills and object-control skills [17]. Locomotor MC (LC) involves proficiency in moving within space, such as running, galloping, skipping, hopping, sliding, and leaping, and object-control MC (OC) involves manipulating objects, to throw, catch, bounce, kick, strike, and roll [18]. There are relatively few studies which report the levels of MC in British preschoolers, although one study has concluded that the levels of MC are low in this population [19].

Despite a dearth of studies examining MC in British preschoolers, other work in Australia has reported that overall preschoolers were not sufficiently active for health, and females are more proficient in LC than males [20]. The aforementioned work [20] also reported that the different aspects of MC were related to PA in differing ways; OC is more strongly associated with MVPA in males, compared to LC, which is more strongly associated with MVPA in females compared to males. For urbanized British preschoolers, overall MC and LC were associated with MVPA, and OC was associated with both MVPA and TPA; males were significantly more active than females, and more proficient in OC [21].

Preschoolers have consistently been identified as undertaking insufficient PA for health [8,10,11,12,13]. Associations between PA and MC have been identified in preschoolers [20,21], but establishing current PA levels and the extent to which British preschoolers complete the recommended amount of PA for health with the use of objective and validated measures, and alongside objective MC assessment methods, has yet to be completed. Therefore, the aim of this study was to address this gap and examine the associations between MC and objectively measured PA, and its relationship to weight-status in British preschool aged children. 

## 2. Materials and Methods 

### 2.1. Participants

Following institutional ethics approval (P31810; 16 February, 2015; Ethics Committee Coventry University), informed parental consent, and child assent, a convenience sample of 177 healthy preschool aged participants (males 54.1%; 4.28 ± 0.74 years) from state funded childcare provisions within the Coventry and Warwickshire area was recruited. Schools and preschools that were recruited were from varied socio-economic backgrounds and participants all attended school or preschool for a minimum of 15 h week. Data collection occurred in the spring and summer months of the school term during childcare hours. Children were included in the analysis if both MC assessments and PA assessments were completed. 

### 2.2. Procedures

#### 2.2.1. Anthropometric Measures

Body mass (to the nearest 0.1 kg) and stature (to the nearest 0.1 cm) were measured objectively by trained researchers using digital scales (SECA Instruments, Ltd, Hamburg, Germany) and a portable stadiometer (SECA Instruments, Ltd, Hamburg, Germany). Body mass index (BMI, kg/m^2^) was calculated, and each child had their weight-status classified as either healthy (HW: 1), or overweight or obese (OW: 2), based on International Obesity Task Force cut-points [22]. 

#### 2.2.2. Motor Competence

MC was assessed using the Test of Gross Motor Development-2 (TGMD-2) [23]. Six locomotor (run, jump, hop, leap, gallop, and slide) and six object control (catch, throw, kick, bounce, strike, and roll) skills were assessed. Each skill comprised three to five components, and skill mastery on the TGMD-2 requires each component to be present. Video recordings of each skill (Sony video camera, Sony, UK) were edited into single-film clips of individual skills with Quintic Biomechanics analysis software v21 (Quintic Consultancy Ltd, Sutton Coldfield, UK). Children completed the TGMD-2 in either school facilities. Each skill was described and demonstrated once by a researcher, and each child performed each skill twice. During analysis, each skill was marked by its individual components as successful (marked as 1) or unsuccessful (marked as 0), and totaled with both trials to give a total skill score. Scores were summed from two trials to create a total overall raw score (scored 0–96) following the recommended TGMD-2 test administration guidelines [23]. The skills identified as LC and OC were grouped together according to subtest scores (LC scored 0–48; OC scored 0–48) and the summing of these gave an overall MC score. All analyses were completed by two trained researchers. Intra- and inter-reliability was established for MC assessments within 15% of the final data set. Intra-rater reliability across MC, LC, and OC showed 0.95, 0.93, and 0.81 agreement, respectively. Inter-rater reliability showed 0.71, 0.90, and 0.81 agreement across MC, LC, and OC, respectively. 

#### 2.2.3. Physical Activity

PA was determined using wrist worn triaxial accelerometry (GENEActiv Activeinsights, Cambridge, UK). Accelerometers were worn for 4 consecutive days on the child’s dominant hand [24] during all waking hours, except for water based activities to prevent skin irritation when drying. A sampling frequency of 100Hz was employed with data collected in 1 s epochs. The accelerometer in question has demonstrated acceptable reliability and validity as a PA measure in children [24,25]. Valid wear time was defined as a minimum of four consecutive days with two weekend days, with at least 10 h of data recorded between 6 am and 10 pm. Non-wear time was defined as 20-minute windows of consecutive zero or non-zero counts [26].

PA was classified as sedentary, light, or moderate-to-vigorous in nature using the Roscoe et al. [27] cut-points, as these are the only validated cut-points for British preschool aged children. This data was then used to determine children who did and did not meet the TPA recommendations, the MVPA recommendations, and the combined recommendations of both TPA and MVPA (WHO, 2010). For assessment of TPA ≥180 min a day, MVPA ≥60 min for zero-to-five-year olds is considered appropriate [2,6,7], and children were classified as sufficiently active if this requirement was met.

### 2.3. Statistical Analysis

Descriptive statistics were calculated by all sex groups and weight-status, and reported as means (± SD). Percentages of children that completed each PA recommendation were calculated, as well as associations between BMI, MC, and PA, which were examined via Pearson’s product moments correlations. Sex and weight-status differences in PA and MC were examined by independent *t*-tests. Chi-squared analysis was used to identify differences in MC between those who did and did not meet PA recommendations. A series of analysis of covariance (ANCOVA) were conducted to compare MC scores between children who did and did not complete PA recommendations, whilst controlling for BMI. Data was analyzed using IBM SPSS Statistics Version 21 (IBM Corporation, New York, NY, USA), with statistical significance set at *p* < 0.05.

## 3. Results

### 3.1. Overview

A total of 94% (*n* = 166) of the original 177 children completed all MC and PA assessments, and were therefore included in final analysis. Means (± SD) are presented, along with descriptive statistics and PA recommendation completion percentages, for all participants, male, female, healthy weight, and overweight children (see Table 1). Males were more active (TPA and MVPA) and were more proficient in OC than females. Overweight children had higher TPA and were more capable in overall MC and LC skills. Males were more likely to complete TPA recommendations, however females were more likely to complete MVPA recommendations. Healthy weight children were more likely to complete TPA, MVPA, and combined PA recommendations.

### 3.2. Associations

Significant associations were found between PA and MC (Table 2). Specifically, between TPA and overall MC (*p* = 0.001), TPA and OC (*p* = 0.001), MVPA and overall MC (*p* = 0.001), and MVPA and OC (*p* = 0.001).

### 3.3. Differences

No significant sex-differences were identified in TPA (*p* = 0.267), MVPA (*p* = 0.718), overall MC (*p* = 0.908), LC (*p* = 0.221), and OC (*p* = 0.342). Similarly, there were no significant differences in the number of males compared to females that completed TPA recommendations (*p* = 0.544), completed MVPA recommendations (*p* = 0.199), or completed combined PA recommendations (*p* = 0.901).

There were no significant weight-status differences in TPA (*p* = 0.936), MVPA (*p* = 0.886), overall MC (*p* = 0.599), LC (*p* = 0.991), and OC (*p =* 0.438). Additionally, there were no differences in the number of children that completed TPA recommendations (*p* = 0.836), completed MVPA recommendations (*p* = 0.636), and completed combined PA recommendations (*p* = 0.689), between healthy weight and overweight children.

When controlling for BMI, overall MC was significantly higher in children who completed TPA recommendations (*p* = 0.008), who completed MVPA recommendations (*p* = 0.014), and who completed combined PA recommendations (*p* = 0.014), than children who did not (see Table 3). Furthermore, LC was significantly higher in children who completed TPA recommendations (*p* = 0.050) than those who did not, and OC was significantly higher in children than completed MVPA recommendations (*p* = 0.003).

## 4. Discussion

This study examined associations between MC and objectively measured PA, and the relationship to weight-status, in British preschool aged children. Importantly, the current study also examined the proportion of children who completed the recommended levels of total PA and MVPA for health. Within this cohort, 80.30% of children achieved TPA recommendations, 89.39% achieved MVPA recommendations, and 75.76% achieved both PA recommendations. This data suggests that most British preschool children in this sample were sufficiently active for health. In agreement with previous literature, there were no significant sex-differences in TPA, MVPA, or combined PA recommendation compliance [12]. However, it is important to note that males were more likely to complete TPA recommendations and females were more likely to complete MVPA recommendations. There were also no significant weight status differences in PA.

Positive associations between TPA and overall MC, TPA and OC, MVPA and overall MC, and MVPA and OC, were identified consistent with previous literature [5,20,21]. There were no significant sex-differences identified in overall MC, LC, or OC, which is congruent with previous literature [19,20,21]. The lack of significant sex-differences can be explained by the age and stage of the cohort, as they are in early childhood and MC is yet to mature [5]. Additionally, there were no significant differences in MC between overweight and normal weight children. Stodden et al. [5] suggests that MC is a precursor to PA; as there are no sex or weight-status differences in PA, as expected, MC was similar.

This is the first study to quantify the level of PA undertaken by British preschoolers using PA cut-points validated with British preschool aged children [27]. This is coupled with use of an objective and valid method for assessing MC [23]. This study therefore addresses limitations of prior studies examining the same topic in British preschoolers [8,12,13]. In the current study, children who completed PA (TPA, MVPA, and combined) recommendations for health-related benefits were significantly more proficient in overall MC. Children that achieved ≥180 min of any PA per day had significantly better LC scores than those that did not. Additionally, children that accomplished ≥60 min of MVPA per day had significantly better OC scores. These findings provide a potential strategy for intervention to increase PA in children, and ultimately to reduce childhood obesity. To improve completion of ≥180 min of TPA for health, improving LC may help, and improving OC should improve the completion of ≥60 min of MVPA for health, and vice versa. Given there were no significant sex or weight status differences in the current study, the results presented here suggest that there is no need for interventions to be sex or weight status specific.

The strengths of this study include the use of a sensitive process-orientated measure of MC (TGMD-2) [23], and objective and validated measurement of PA in children aged three to five years. Additionally, the TGMD-2 is a commonly used and investigated MC assessment measure present in a large proportion of MC studies [28]. It focused on three-to-five-year old children, an understudied but possibly significant age group, particularly given the closeness to adiposity rebound in children. With a 93% compliance rate, this study is still not without limitations. The cross-sectional design, which means causality cannot be inferred, may have underestimated PA, as accelerometers were removed during water-based activities, which included swimming; however, the accelerometers used have been identified as a valid method to capture PA [24].

This study found positive associations between PA and MC in British preschoolers. These associations are particularly strong with TPA and overall MC and LC, and MVPA with overall MC and OC. This study suggests that good motor competence is an important correlate of children meeting physical activity guidelines for health. The novel finding is MC was significantly different between children that met PA recommendations for health and children that did not. Overall MC was significantly better in children that completed all PA recommendations (TPA, MVPA, and combined). Children were significantly more proficient in LC if they completed ≥180 min of TPA per day, and OC was significantly better in children that completed ≥60 min of MVPA per day. This finding provides more detailed understanding of the relationship between PA and MC, and can be used in the development of impactful interventions to improve MC and PA in this age group. It is possible that different aspects of MC may be required to promote PA and vice versa, and may be used to encourage an active lifestyle in young children. However, longitudinal research is needed to better understand causal relationships between MC, PA, and weight-status, but the findings from the current study can be used to inform the design of developmentally appropriate interventions targeting PA and MC for effective preventative medicine strategies to be initiated to improve PA and MC, and reduce obesity, in early childhood.

## Figures and Tables

**Table 1:** Descriptive values of BMI, Physical Activity, and Motor Competence.

	All *n* = 166	Males *n* = 91	Females *n* = 75	HW *n* = 146 53% Male	OW *n* = 20 75% Male
**Age (years)**	4.28 ± 0.74	4.34 ± 0.74	4.21 ± 0.73	4.27 ± 0.75	4.33 ± 0.64
**BMI (kg/m^2^)**	16.11 ± 1.65	16.27 ± 1.78	15.93 ± 1.47	15.65 ± 1.11	19.09 ± 1.42
**TPA (mins)**	279.65 ± 118.33	289.95 ± 119.99	266.90 ± 116.00	279.36 ± 116.48	282.07 ± 137.77
**MVPA (mins)**	238.69 ± 101.87	248.06 ± 104.85	227.10 ± 97.71	239.18 ± 99.74	234.59 ± 122.61
**Overall MC (out of 96)**	45.73 ± 12.07	45.73 ± 13.01	45.88 ± 10.75	43.46 ± 12.19	44.29 ± 11.59
**LC (out of 48)**	26.80 ± 7.60	26.10 ± 8.01	27.80 ± 6.95	26.80 ± 7.83	26.82 ± 6.13
**OC (out of 48)**	18.93 ± 8.30	19.53 ± 9.04	18.08 ± 7.13	19.16 ± 8.15	17.47 ± 9.29
**% that complete TPA recommendations**	80.30	82.19	77.97	61.69	45.83
**% that complete MVPA recommendations**	89.39	86.30	93.22	68.83	50.00
**% that complete combined PA recommendations**	75.76	75.34	76.27	58.44	41.67

Note: MC, motor competence; LC, locomotor motor competence; OC, object-control motor competence; PA, physical activity; TPA, total PA; MVPA, moderate-to-vigorous PA; BMI, body mass index; HW, healthy weight; OW, overweight or obese.

**Table 2:** Descriptive values of BMI, PA, and MC.

	BMI	TPA	MVPA	Overall MC	LC	OC
**BMI**		R = 0.018	R = 0.012	R = −0.043	R = −0.045	R = −0.022
**TPA**			R = 0.733 **	R = 0.402 **	R = 0.170	R = 0.386 **
**MVPA**				R = 0.376 **	R = 0.152	R = 0.367 **
**Overall MC**					R = 0.734 **	R = 0.783 **
**LC**						R = 0.152
**OC**						

Note: MC, motor competence; LC, locomotor motor competence; OC, object-control motor competence. ** Correlation is significant at the 0.01 level (2-tailed). * Correlation is significant at the 0.05 level (2-tailed).

**Table 3:** Mean (± SD) MC in children who met and did not meet PA recommendations.

	TPA Recommendations		MVPA Recommendations		Combined PA Recommendations	
	Met	Not Met	Met	Not Met	Met	Not Met
**Overall MC**	45.99 ± 11.16 **	38.47 ± 10.55	45.17 ± 10.89 *	35.14 ± 13.98	45.88 ± 11.20 *	39.20 ± 10.77
**LC**	26.70 *± 6.86 *	23.37 ± 7.52	26.00 ± 7.19	25.86 ± 6.34	26.65 ± 6.90	23.70 ± 7.47
**OC**	19.29 ± 8.35	15.11 ± 9.53	19.17 ± 8.09 **	9.29 ± 11.31	19.23 ± 8.40	15.50 ± 9.44

Note: MC, motor competence; LC, locomotor competence; OC, object-control competence. ** Correlation is significant at the 0.01 level (2-tailed). * Correlation is significant at the 0.05 level (2-tailed).
